# Clinical outcomes in primary scalp angiosarcoma

**DOI:** 10.3892/ol.2020.12025

**Published:** 2020-08-26

**Authors:** Yong Zhang, Yanwen Yan, Ming Zhu, Cheng Chen, Nanhang Lu, Fazhi Qi, Jiaqi Liu

Oncol Lett 18: 5091-5096, 2019; DOI: 10.3892/ol.2019.10886

After the publication of the above article, the authors have realized that Figs. 8 and 9 in their paper were presented incorrectly; essentially, the red lines plotted to show the nodular data in these Figures should not have been drawn to meet the *x* axis. The corrected versions of Figs. 8 and 9 are shown opposite. Note that the revisions made to these figures do not affect the overall conclusions reported in the paper. The authors apologize to the Editor of *Oncology Letters* and to the readership for any inconvenience caused.

## Figures and Tables

**Figure 8.**
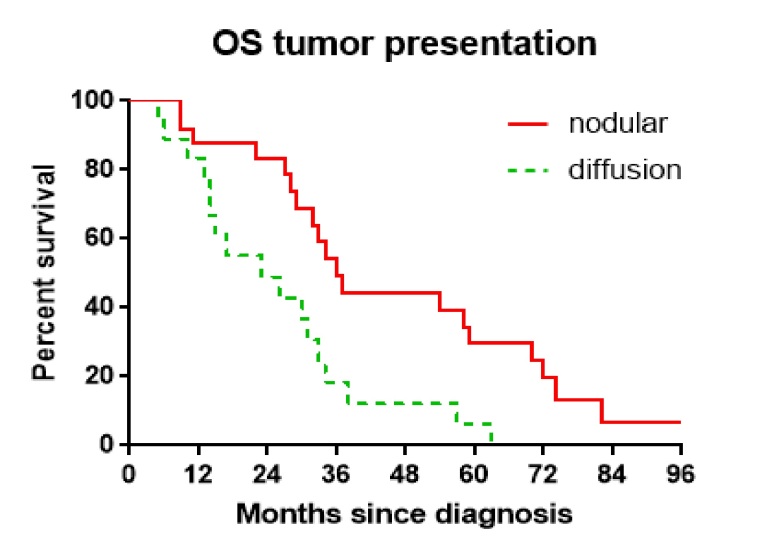
Overall survival comparison based on tumor presentation. Wilcoxon test, P=0.007.

**Figure 9.**
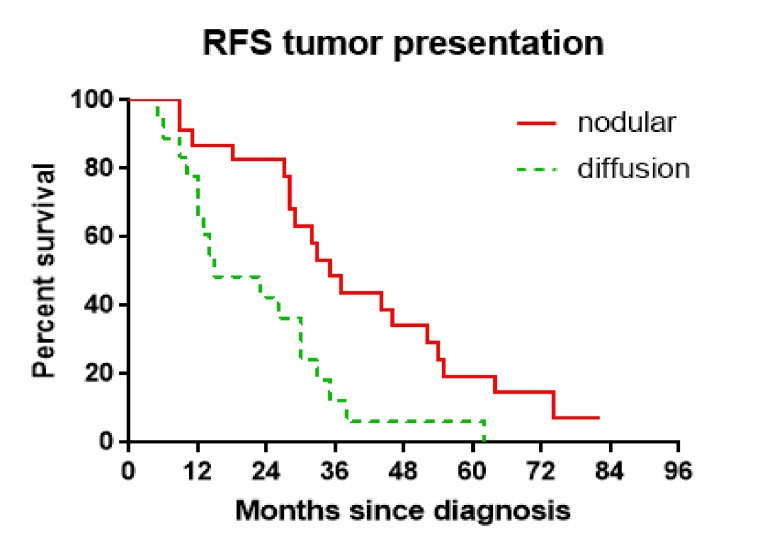
Recurrence-free survival comparison based on tumor presentation. Wilcoxon test, P=0.0058.

